# Complete Genome Sequence of Escherichia coli MT102, a Plasmid-Free Recipient Resistant to Rifampin, Azide, and Streptomycin, Used in Conjugation Experiments

**DOI:** 10.1128/MRA.00383-19

**Published:** 2019-05-16

**Authors:** Adam Valcek, Søren Overballe-Petersen, Frank Hansen, Monika Dolejska, Henrik Hasman

**Affiliations:** a CEITEC VFU, University of Veterinary and Pharmaceutical Sciences Brno, Brno, Czech Republic; b Department of Biology and Wildlife Diseases, Faculty of Veterinary Hygiene, University of Veterinary and Pharmaceutical Sciences Brno, Brno, Czech Republic; c Department of Bacteria, Parasites and Fungi, Statens Serum Institut, Copenhagen, Denmark; University of Rochester School of Medicine and Dentistry

## Abstract

We present here the complete genome sequence of Escherichia coli MT102, which is resistant to rifampin, azide, and streptomycin and is used as a recipient in plasmid transfer experiments. The sequence will be utilized for chromosomal read removal in plasmid sequence analyses obtained from transconjugants within this strain and in comprehensive genetic studies.

## ANNOUNCEMENT

Escherichia coli MT102 is an *araD139* (*ara-leu*)*7697* Δ*lac thi hsdR* derivate of E. coli K-12 substrain MC1000 (1, 2) and was constructed by Mogens Trier Hansen at Novo-Nordisk. E. coli MT102 is resistant to sodium azide, rifampin, and streptomycin due to missense mutations in the *secA* (3), *rpoB* (4), and *rpsL* (5) genes, respectively. This strain has been used in several studies characterizing mobile genetic elements carrying antimicrobial genes (6–8) and studies requiring a plasmid-free host (9, 10). E. coli MT102 was grown on MacConkey agar plates supplemented with rifampin (25 mg/liter) and sodium azide (25 mg/liter) overnight at 37°C. Bacterial genomic DNA for short-read Illumina sequencing was extracted using the DNeasy blood and tissue kit (catalog number 69506; Qiagen, Hilden, Germany), and a sequencing library was prepared using the Nextera XT kit (catalog number FC-131-1096; Illumina, San Diego, CA). Short reads were obtained by 2 × 250-bp paired-end MiSeq sequencing (Illumina), yielding 3,312,280 reads. Reads were trimmed using Trimmomatic v0.36 (illuminaclip:TruSeq3-PE.fa:2:30:10 leading:3 trailing:3 slidingwindow:4:15 minlen:36) (11) in order to remove adaptor residues and low-quality (Q ≤ 20) ends. For the long reads, DNA was prepared using the Agencourt Genfind v2 kit (Beckman Coulter, Brea, CA) with a DynaMag-2 magnet (Thermo Fisher, Waltham, MA, USA). Libraries were prepared with the 1D ligation barcoding kit (catalog numbers SQK-LSK108 and EXP-NBD103; ONT, Oxford, United Kingdom) and sequenced in an R9.4 flow cell (catalog number FLO-MIN106) with a MinION Mk1B device (ONT), yielding 16,561 reads consisting of 138,366,031 bp in total, with an *N*_50_ read length of 14,053 bp. The fast5 reads were base called, demultiplexed, and converted to fastq format using Albacore v2.0.2 (ONT) with default settings. The adaptor sequences were removed using Porechop v0.2.2 (12) with default settings. Hybrid assembly of long and short reads was performed using Unicycler v0.4.0 (13), with default settings, and resulted in one circular contig of the bacterial chromosome. The length of the contig was 4,548,459 bp. The coverages of Illumina reads were 174× and 25× of the long reads from MinION. The short Illumina reads were mapped to the chromosomal contig using the “Map to reference” option in Geneious 9.0.5 (Biomatters Ltd., Auckland, New Zealand) and called variants using “Find variations/SNPs…” based on the mapping for manual error correction, both with default settings. Moreover, MinION reads longer than 10 kb were mapped to the chromosomal contig to double-check the consensus sequence. Due to the manual error correction, the size of the final chromosomal sequence was increased when 489 bp was added.

The genome was annotated using NCBI Prokaryotic Genome Annotation Pipeline (14). The complete genome of E. coli MT102 consists of 4,548,948 bp, with a GC content of 50.7%, 4,288 coding sequences, 86 tRNAs, 22 rRNAs, and 15 noncoding RNAs (ncRNAs). The strain belongs to sequence type 10, which was identified using MLST-2.0 (15) by the Center for Genomic Epidemiology (CGE; DTU, Kongens Lyngby, Denmark).

## 

### Data availability.

The complete genome sequence of E. coli MT102 and raw data have been deposited in GenBank under accession number CP034953 and SRA accession numbers SRX5367507 and SRX5367508.
